# Cementing mussels to oysters in the pteriomorphian tree: a phylogenomic approach

**DOI:** 10.1098/rspb.2016.0857

**Published:** 2016-06-29

**Authors:** Sarah Lemer, Vanessa L. González, Rüdiger Bieler, Gonzalo Giribet

**Affiliations:** 1 Museum of Comparative Zoology, Department of Organismic and Evolutionary Biology, Harvard University, 26 Oxford Street, Cambridge, MA 02138, USA; 2 Department of Invertebrate Zoology, National Museum of Natural History, Smithsonian Institution, Washington, DC 20013, USA; 3 Integrative Research Center, Field Museum of Natural History, 1400 South Lake Shore Drive, Chicago, IL 60605, USA

**Keywords:** phylogenomics, genome, phylogeny, Mollusca, Bivalvia, evolutionary rate

## Abstract

Mussels (Mytilida) are a group of bivalves with ancient origins and some of the most important commercial shellfish worldwide. Mytilida consists of approximately 400 species found in various littoral and deep-sea environments, and are part of the higher clade Pteriomorphia, but their exact position within the group has been unstable. The multiple adaptive radiations that occurred within Pteriomorphia have rendered phylogenetic classifications difficult and uncertainty remains regarding the relationships among most families. To address this phylogenetic uncertainty, novel transcriptomic data were generated to include all five orders of Pteriomorphia. Our results, derived from complex analyses of large datasets from 41 transcriptomes and evaluating possible pitfalls affecting phylogenetic reconstruction (matrix occupancy, heterogeneity, evolutionary rates, evolutionary models), consistently recover a well-supported phylogeny of Pteriomorphia, with the only exception of the most complete but smallest data matrix (*Matrix 3*: 51 genes, 90% gene occupancy). Maximum-likelihood and Bayesian mixture model analyses retrieve strong support for: (i) the monophyly of Pteriomorphia, (ii) Mytilida as a sister group to Ostreida, and (iii) Arcida as sister group to all other pteriomorphians. The basal position of Arcida is congruent with its shell microstructure (solely composed of aragonitic crystals), whereas Mytilida and Ostreida display a combination of a calcitic outer layer with an aragonitic inner layer composed of nacre tablets, the latter being secondarily lost in Ostreoidea.

## 1. Introduction

Mussels (the members of the superfamily Mytiloidea) are a ubiquitous and common group of bivalves and a main source of protein for humans and non-human animals alike. Despite being the dominant organisms in many littoral, shallow sub-littoral, deep-sea hydrothermal vent and cold seep ecosystems [1]—including rocky and sediment shores on open coasts and in estuaries and marshes [2]—the position of mussels in relation to other pteriomorphian bivalves remains unresolved (e.g. [3]). Because of their economic and ecological importance, mussels have been the subject of considerable research effort. Taxonomically, Mytiloidea is a diverse group of pteriomorphian bivalves recognized by characteristics of shell form, hinge and mussel attachment scars [4].

Mytilidae, by most authors considered the only family in the superfamily Mytiloidea and the order Mytilida (but see Carter [5]), is an ancient group with roots extending to the Devonian [6]. It comprises eight recognized extant subfamilies [7,8], with approximately 400 species worldwide [9], probably constituting the largest family of bivalves in number of species. Like mussels, in particular, bivalves of the subclass Pteriomorphia in general form a commercially important clade with species harvested and cultured worldwide both for food (e.g. oysters, scallops, ark shells), and ornament industry (pearl oysters). They are characterized by a large variability in ligament structure and in shell size, shape and composition. Some shells are constituted of aragonite tablets only (e.g. Arcida), others of calcite crystals (e.g. Ostreoidea), whereas some species present a shell that has both crystal forms of calcium carbonate, calcite and aragonite (e.g. Mytilida, Pectinida, Limida, Pinnoidea and Pterioidea) [10]. Shell composition is important because most pteriomorphians are sessile as adults, their calcified shells being the only mechanism against predation and desiccation [11]. In addition, understanding the evolution of shell formation can help assess the impacts of ocean acidification on bivalves.

Pteriomorphia appears in the fossil record in the Early Ordovician where mytiloid, arcoid and pterioid forms are recognized. These groups experienced periods of radiating diversification in the Late Devonian, as well as in the beginning and the end of the Mesozoic [12]. The diversity of this group reflects several adaptive radiations and the shell forms have attracted the interest of many palaeontologists and neontologists (e.g. [13–21]). The multiple adaptive radiations have rendered phylogenetic classifications difficult owing to convergence and/or parallel evolution at various levels [22]. Although the most recent phylogenies agree on the monophyly of Pteriomorphia, uncertainty remains regarding the internal relationships of the majority of pteriomorphian clades (families and superfamilies), despite considerable phylogenetic (e.g. [16,18,23–27]) and even recent phylogenomic [28] efforts.

Pteriomorphia comprises five widely recognized extant orders (Arcida, Limida, Mytilida, Ostreida and Pectinida) [27]. Among the most unstable results in bivalve phylogeny are the relative positions of Arcida and Mytilida ([3,29]). As one of the most diverse and studied bivalve groups, it is unsettling that to this day relationships among pteriomorphians in general and mytiloids in particular remain unresolved, impinging on future studies aiming at exploring extinction, diversification and biogeographic patterns of this group, including dating and inference of the evolution of lineages through time. With the aim of improving resolution within Pteriomorphia, we generated a novel Illumina-based dataset to evaluate the internal branching patterns of this clade from a fresh perspective and infer on the evolution of shell microstructure within pteriomorphians. For this, we sequenced, assembled and analysed 12 new pteriomorphian transcriptomes and combined them with 13 additional transcriptomes previously generated by us [28], and 22 transcriptomes and one genome from publicly available data, for a total of 41 pteriomorphian samples and eight outgroups. The taxonomic sampling used here reflects what is now acknowledged to be the span of pteriomorphian diversity.

## 2. Material and methods

### (a) Taxon sampling, cDNA library construction and next-generation sequencing

We sequenced cDNA from 12 pteriomorphian specimens using an Illumina HiSeq 2500 platform and combined these with 35 published transcriptomes and one genome, including 13 libraries previously sequenced in our laboratory [28]. Information about the sampled specimens can be found in the electronic supplementary material, table S1 and in the MCZ online collections database (http://mczbase.mcz.harvard.edu). All tissues were collected fresh and immediately flash frozen in liquid nitrogen or fixed in RNA*later*^®^ (Life Technologies, Carlsbad, CA, USA) and stored at −80°C. Total RNA was extracted using TRIzol (Life Sciences) and purification of mRNA was performed using the Dynabeads (Invitrogen) following the manufacturer's instructions and as described in [28]. For each sample, quality of mRNA was assessed with a picoRNA assay in an Agilent 2100 Bioanalyzer (Agilent Technologies) and quantity measured with an RNA assay in a Qubit fluorometer (Life Technologies).

All cDNA libraries were constructed using the PrepX mRNA kit for Apollo 324 (Wafergen). Libraries were sequenced on the Illumina HiSeq 2500 platform with paired-end reads of 150 bp at the FAS Center for Systems Biology at Harvard University, after their concentration and quality were assessed.

### (b) Transcriptome assembly

All reads generated for this study are deposited in the National Center for Biotechnology Information Sequence Read Archive (NCBI-SRA; electronic supplementary material, table S1). Each sample, except for the genome of *Pinctada fucata*, was prepared as in [28], as detailed in the electronic supplementary material, S2. De novo assemblies were conducted for each sample with Trinity r2014-04-13 [30,31] using paired read files and default parameters except for ‘--path_reinforcement_distance 50’, which seems to produce slightly better assemblies, with higher N50 values and longer contigs. Reduction of redundant reads, peptide prediction and peptide filtration were conducted as in [28] (detailed in the electronic supplementary material, S2).

### (c) Orthology assignment and matrix construction

Orthology assignment for the dataset assemblies was performed using stand-alone OMA v. 0.99z.2 [32,33] (detailed parameters in the electronic supplementary material, S2). Three initial data matrices following occupancy thresholds [34] were generated for phylogenetic analyses: the first one, *Matrix 1*, targeting a minimum gene occupancy of 50%, was constructed by selecting the OMA orthogroups present in 24 or more taxa (1205 orthogroups). *Matrix 2* includes orthogroups present in 36 or more taxa (gene occupancy greater than 75%; 277 orthogroups). *Matrix 3* was constructed by selecting the orthogroups found in 44 or more taxa (greater than 90% gene occupancy; 51 orthogroups). The orthogroup selection based on minimum taxon occupancy was executed using a custom Python script (all scripts and data matrices are available online at https://dataverse.harvard.edu/dataverse/Pteriomorphia_phylogenomics). Alignments were generated for each matrix using MUSCLE v. 3.6 [35] (details in the electronic supplementary material, S2).

**Figure 1.**
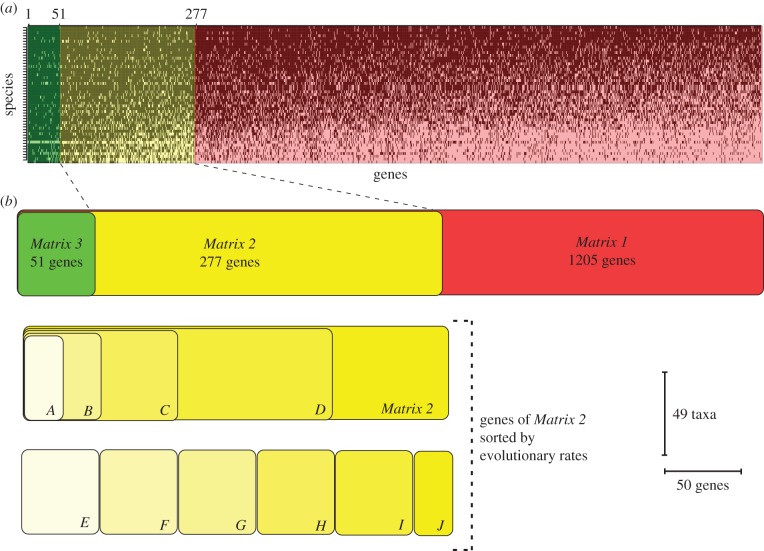
(*a*) Gene occupancy representation per species with maximum occupancy towards the top left. A light cell indicates a non-sampled gene. *Mytilus galloprovincialis* is the best-represented species, whereas *Saccostrea palmula* is the worst-represented one. *Matrix 1* is represented in red, *Matrix 2* in yellow and *Matrix 3* in green. (*b*) Schematic of the 13 matrices generated for the phylogenetic, per cent pairwise identity and compositional heterogeneity analyses. Colour codes are as in (*a*). (Online version in colour.)

In order to assess the effects of rate of molecular evolution and heterotachy on tree topology, 10 additional matrices were constructed by selecting subsets of *Matrix 2* based on evolutionary rate, for which per cent pairwise identity was employed as a proxy (see figure 1 and the electronic supplementary material, S2, for details). This method was chosen to approximate evolutionary rate because it is agnostic to tree topology. Accumulated conservation values were generated for each locus using Trimal 1.2b (-sct flag). Matrices were produced as follows: (i) *Matrices A–D* were constructed with incremental addition of loci to produce four matrices (figure 1). This strategy has been used in previous phylogenomic analyses [36–38], but masks the possible contribution of the fastest evolving genes, as the slowest ones are always used. In order to investigate the contribution of the different blocks of evolutionary rates in the absence of the other genes, we designed another strategy: *Matrices E–J* were constructed by parsing 50 loci matrices and one 27 loci matrix, with no addition (figure 1). Compositional heterogeneity among taxa and within each orthogroup can affect phylogenetic results and lead to incorrect tree reconstructions. In order to discern if this was the case in our dataset we used the package BaCoCa v. 1.1r [39] to estimate relative composition frequency variability (RCFV) in *Matrix 2*.

### (d) Phylogenetic analyses

All matrices, but *Matrix 1*, were analysed using maximum-likelihood inferences conducted by PhyML-PCMA [40]. PhyML-PCMA estimates a model through the use of a principal component (PC) analysis. We selected 10 PCs in the PhyML-PCMA analyses. Because of its large size and the intense computation required by PhyML-PCMA, *Matrix 1* was analysed with ExaML v. 3.0 [41] (see the electronic supplementary material, S2, for detailed parameters).

The initial three matrices (*Matrices 1*, *2* and *3*) were also analysed using Bayesian inference with ExaBayes v. 1.21 with openmpi v. 1.64 ([42]; see the electronic supplementary material, S2, for detailed parameters). Additional Bayesian tree searches were also conducted for *Matrices 2* and *3* in PhyloBayes MPI v. 1.4e [43] using the site-heterogeneous CAT-GTR model of evolution [44]. Four independent Markov chain Monte Carlo (MCMC) runs were conducted for 2196–3187 cycles. The initial cycles in each MCMC run were discarded as burn-in and determined using the ‘tracecomp’ executable. Convergence was assessed using the ‘bpcomp’ executable and chains were considered to have converged when the maximum bipartition discrepancies (maxdiff) across a minimum of two independent chains reached 0.2.

Finally, because *Matrix 3* showed inconsistent tree topologies across analyses (see Results), we conducted additional tree searches after partitioning this matrix into genes, using RAxML 7.7.5 [45] with the GAMMA model of rate heterogeneity, the WAG protein substitution model and 1000 bootstraps.

To test for putative gene incongruence, we inferred individual gene trees for each orthogroup included in each of the three initial matrices (*Matrices 1*, *2* and *3*) using RAxML 7.7.5 [45] and SuperQ v. 1.1 [46] (see the electronic supplementary material, S2, for detailed parameters).

## 3. Results

### (a) Phylogenetic relationships based on the three main matrices

The number of sequence reads, used reads, accession numbers, contigs, and other values to assess the quality of the assembled transcriptomes, can be found in the electronic supplementary material, table S1. Orthology assessment of this 49-taxon dataset with the OMA stand-alone algorithm recovered 149 182 orthogroups. The three super-matrices generated yielded 1205 (*Matrix 1*: occupancy of more than 50%; 316 219 aa), 277 (*Matrix 2*: occupancy of more than 75%; 64 318 aa) and 51 (*Matrix 3*: occupancy of more than 90%; 11 066 aa) orthologs*,* respectively.

**Figure 2.**
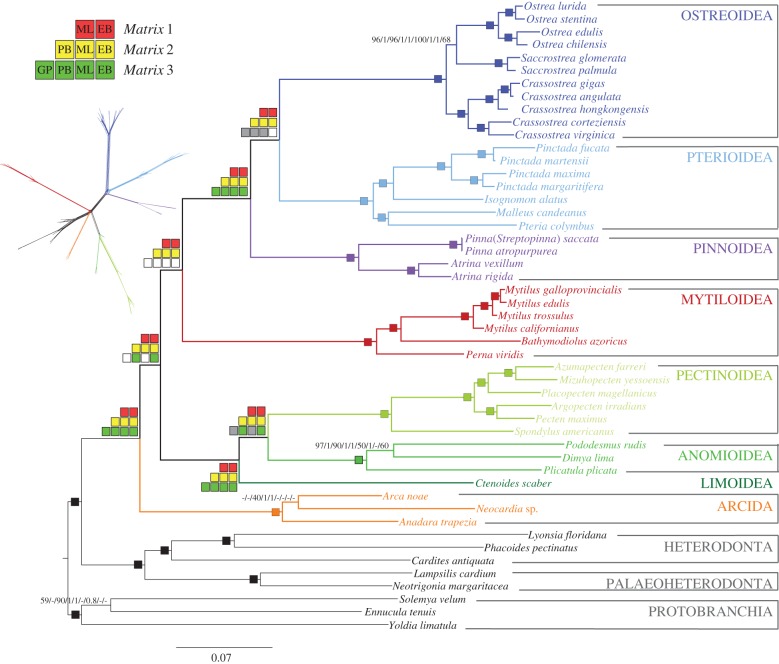
Phylogenetic hypothesis based on *Matrix 2* analysed in PhyML-PCMA (Ln *L* = −1 132 164.323494) with support values plotted as follows: checked boards in major deep nodes represent nodal support for the different analyses in *Matrix 1* (red), *Matrix 2* (yellow) and *Matrix 3* (green). PhyML-PCMA and ExaML are abbreviated as ML, ExaBayes: EB, PhyloBayes: PB, and RAxML with gene partitions: GP. Filled squares indicate nodal support values higher than 99% BS (ML) and a pp of 0.99 or higher (EB). Grey squares indicate lower nodal support and white squares indicate unrecovered nodes in the specified analysis. Single squares on internal nodes indicate maximum support in all six analyses. Lower nodal support values of internal nodes are reported as follows: *Matrix 1* ML/*Matrix 1* EB/*Matrix 2* ML/*Matrix 2* EB/*Matrix 2* PB/*Matrix 3* ML/*Matrix 3* EB/*Matrix 3* PB/*Matrix 3* GP. Pteriomorphian families are represented in different shades of colour. Top left: supernetwork representation of quartets derived from the individual ML gene trees for *Matrix 2*. The same colour scheme is applied to all figures. (Online version in colour.)

All the phylogenetic analyses conducted on all three matrices revealed well-supported consistent topologies for most pteriomorphian superfamilies (figure 2). The maximum-likelihood (ML: PhyML, ExaML and RAxML) and Bayesian (ExaBayes and PhyloBayes) phylogenetic analyses conducted on the three main matrices (*Matrix 1*, *Matrix 2*, *Matrix 3*) recovered the monophyly of all five pteriomorphian orders, superfamilies and families, except for Arcoidea, which was not recovered as monophyletic and included Limopsoidea in all analyses conducted with *Matrix 2* (figure 2). Ostreoidea and Pterioidea appeared as sister clades with maximum support (100% bootstrap support (BS) and a posterior probability (pp) of 1.00; for all ML and Bayesian analyses, respectively) with Pinnoidea as their sister group in all analyses except in analyses conducted with *Matrix 3*, mostly refuting prior morphology-based hypotheses (e.g. [47]; figure 4). The relationships among superfamilies of Pectinida were consistent and well supported in all analyses: Anomioidea and Pectinoidea were sister clades (figure 2). Similarly, Limida was unvaryingly recovered as the sister group to Pectinida with 100% BS or a pp of 1.00 in all analyses and with all the analysed matrices.

The position of Mytilida was consistent and well supported in the five analyses with *Matrices 1* and *2* (figure 2), constituting the sister group to Ostreida with a 99% BS and a pp of 1.00. However, the phylogenetic analyses with *Matrix 3* were inconsistent across methods and placed Mytilida either more basally, as sister group to Arcida (ML and PhyloBayes; 27% BS and pp = 1.00, respectively), as sister group to the clade constituted by Pectinida and Limida (ExaBayes; pp = 0.89), or as a sister group to all pteriomorphians except Arcida (RAxML with gene partitions; 47% BS). The lack of nodal support for this smallest matrix thus indicates that not enough information was available in *Matrix 3* to resolve the position of Mytilida and that more than 51 genes may be needed to resolve this ancient divergence. Nonetheless, Arcida was always recovered at the base of the pteriomorphian tree, either as sister group to all other pteriomorphians, or as sister group to Mytilida, even with *Matrix 3*.

The supernetworks obtained for each matrix using SuperQ v. 1.1, displayed a tree-like structure with relatively long edges and similar topologies as to the concatenated species trees (figure 2; electronic supplementary material, figure SA). All three networks show a long edge leading to the clade formed by Ostreida (*sensu* [27]); however, they also indicated gene conflict for the position of Mytilida with respect to Arcida, and the clade including Limida and Pectinida, especially in the supernetwork for *Matrix 3*. Reticulations were also visible in the supernetwork obtained with *Matrix 3* relative to the position of Pterioidea and gene conflict was detected in more derived nodes within Ostreida, probably reflecting on the poor quality of the *P. fucata* genome (electronic supplementary material, figure SA).

### (b) Concatenation by per cent pairwise identity and compositional heterogeneity

**Figure 3.**
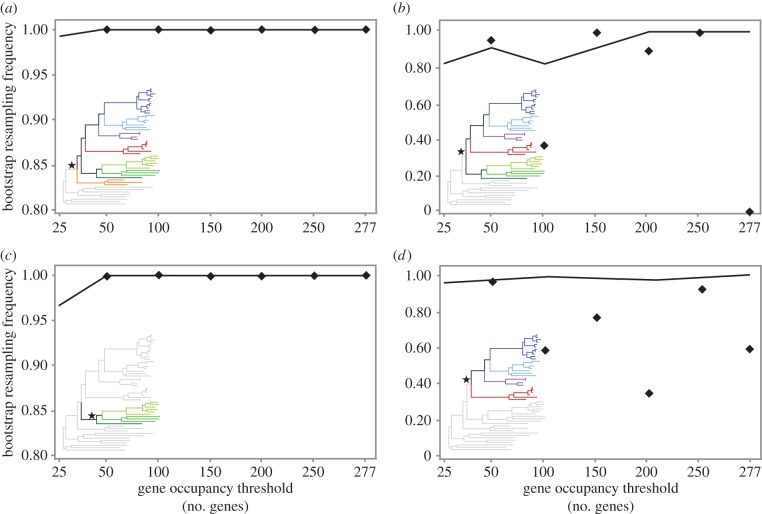
Bootstrap resampling frequency for phylogenetic hypotheses (*a*) monophyly of Pteriomorphia, (*b*) basal position of Arcida (=monophyly of all other pteriomorphians), (*c*) monophyly of Limida + Pectinida, (*d*) Mytilida sister group to Ostreida; inferred from *Matrices A–D* and *Matrix* 2, represented in full line (incremental addition of orthogroups in order of per cent pairwise identity, from most to least conserved); *Matrices E–J*, represented by diamonds (increments of non-additive 50 orthogroups in order of per cent pairwise identity, from most to least conserved). (Online version in colour.)

As the position of Mytilida has been in flux, we focused our analyses on the putative node uniting Mytilida with Ostreida. When evaluating the matrices obtained by sequentially adding genes based on their evolutionary rates (*Matrices A*–*D* and *Matrix 2*; see figure 1 for details), we observed a monotonic trajectory of nodal support increasing from 97% to 100% BS (full line in figure 3*d*). A similar result was obtained for the monophyly of Pteriomorphia and the sister group relationship of Limida and Pectinida (full lines in figure 3*a*,*c*). The nodal support for the basal placement of Arcida, i.e. monophyly of all other pteriomorphians (full line in figure 3*b*), showed a more gradual and fluctuating increase when adding sets of genes with higher evolutionary rates, going from 81% to 100% BS. By contrast, when looking at *Matrices E–J* (no addition; figure 1), we observed a fluctuating trajectory of BS for the sister group relationship of Mytilida and Ostreida, with maximum support obtained with the 50 most conserved genes (96% BS) but thereafter nodal support oscillated between 35 and 93%, with no clear trend (diamonds in figure 3*d*). The support for the basal placement of Arcida also showed some fluctuation: between 90 and 100% BS was found for all but two sets of 50 genes. The second set of fastest evolving genes only displayed 37% BS and the last set of the fastest-evolving 27 genes failed to support the topology altogether and instead placed Arcida as sister group to Pectinida (diamonds in figure 3*b*). The latter placement of Arcida was not retrieved in any of the conducted phylogenetic analyses and probably reflects on the poor ability of a small subset of fast evolving genes to recover deep phylogenetic nodes in Pteriomorphia. The monophyly of Pteriomorphia and the sister group relationship of Limida and Pectinida (diamonds in figure 3*a*,*c*) were supported in all analyses, which found 100% BS for all sets of 50 genes, irrespective of their evolutionary rate.

The RCFV per taxon and per amino acid ranged from 0.0001 to 0.007 (electronic supplementary material, figure SB), indicating compositional homogeneity throughout all of the amino acids and taxa included in *Matrix 2* and thus eliminating possible biases owing to compositional heterogeneity.

## 4. Discussion

### (a) Cementing mussels to oysters

The phylogenetic dataset generated in this study is, to our knowledge, the largest ever gathered to clarify the position of mussels and to test relationships with their closest relatives. As in many other studies, our results support the monophyly of Pteriomorphia with maximum support and this node is found under all analyses. Nevertheless, the tree topologies recovered by the ML and Bayesian analyses of *Matrix 3* (51 genes) recapitulate a recurring phenomenon in molecular phylogenetic studies of Pteriomorphia: the unstable position of Mytilida. A major concern in phylogenetic reconstruction has been the amount of missing data, as they can produce misleading estimates of topology and branch lengths (e.g. [48,49]). However, with the generalized use of transcriptome-based data matrices for phylogenetic reconstruction, large matrices, even if typically incomplete, have shown the ability to reconstruct relationships with strong support [34]. Perhaps, less intuitive has been the finding that in some cases the most complete matrices are not always providing the most accurate reconstructions, even if nearing 100% completeness, a poorly studied phenomenon, but that may be owing to limited amount of available genetic information in the genes selected for completeness [50]. In our case, *Matrix 3* is the only one with conflicting topologies when using different phylogenetic methods. The PhyML and PhyloBayes analyses conducted with *Matrix 3* place Mytilida as the sister group of Arcida, these two constituting the sister group of all remaining Pteriomorphia; whereas the ExaBayes and gene-partitioned RAxML analyses place Mytilida as sister group to the clade formed by Pectinida and Limida or as sister group to all pteriomorphians excluding Arcida, respectively. None of these topologies, however, received significant support. We thus conclude that the position of Mytilida resolved by the two larger matrices, as sister group to Ostreida, is the best corroborated hypothesis; this result is also recovered by several other submatrices of genes sorted by evolutionary rate (*Matrices A–D, E, I*; figures 1 and 3*d*), including slow evolving genes (*Matrices A, B, E*) and fast evolving genes (*Matrices C, D, I*). The fact that several small matrices, composed of different sets of genes, yield different tree topologies probably explains the disparity of phylogenetic results from previous molecular analyses (e.g. [18,24,26,27]).

The position of Arcida has also been unstable throughout a series of molecular data analyses [18,24,26,27] and hardly any morphological analysis has proposed a sister group relationship to all other pteriomorphians, as many authors have placed Mytilida in a more basal position (e.g. [12,16]). Our results, however uniformly place Arcida in a basal position, as sister group to all other pteriomorphians (as in the morphological analyses of Giribet & Wheeler [24,27]), with the exception of two analyses (PhyML and PhyloBayes, *Matrix 3*) that place Mytilida as their sister group—a partially unsupported relationship (27% BS, but pp = 1.00) that had been suggested previously [51]. These findings do not support the hypothesis of Carter *et al*. [5] that arranged Pteriomorphia into two major clades, Mytilomorphi (with Mytilida) and Ostreomorphi (with the other groups treated herein).

### (b) Implications for the evolution of shell microstructure

Early bivalves built aragonite shells, a character retained in Nuculida, Trigoniida, Unionida and most Anomalodesmata [12,52,53]. Pteriomorphians all develop an outer calcitic layer in addition to the inner aragonitic shell layer, with the exception of Arcida, whose shell microstructure still consists of both outer and inner crossed-lamellar layers of aragonite crystals only [10,27]. Since Arcida displays a basal position with respect to other pteriomorphians, this character requires no homoplasy, as arcoids present the plesiomorphic state found in closely related bivalve outgroups. The development of an outer calcitic layer is thought to be a recent adaptation against shell dissolution in cold and undersaturated waters [54,55]. There are two main types of calcite prisms from the viewpoint of the microstructure: simple prismatic or foliated. The simple prismatic microstructure is found in the outer layers of Pterioidea (except for Malleidae), Pinnoidea and Mytilida (the latter has the particularity of also being fibrous) [10,14]. The outer simple calcitic prismatic layer is a flexible structure that allows tight sealing of the two valves, thus better isolating the inner chamber from changing ambient water conditions and minimizes damage from unsuccessful predation. This shell ultrastructure is usually associated with epi- and endo-byssate life-habits (e.g. Mytilida, Pterioidea, Pinnoidea). By contrast, the clades that developed a foliated calcite layer (Pectinida, Ostreoidea and the family Malleidae) gained the opportunity to produce more convex valves with a wide repertoire of ornamentations and new shell morphologies, making this character a key innovation in the adaptive radiation of Pteriomorphia. The foliate microstructure is a derived feature that is more rigid but less resistant to breakage than the simple prismatic structure [56]; clades that adopted this arrangement are more widespread, have higher generic diversity and have repeatedly evolved more derived life-habits, e.g. cementing (Ostreoidea), swimming (Pectinoidea) or free reclining (Anomioidea) [57]. The calcitic outer layer of Limida consists of a combination of calcitic fibrous prismatic and crossed-foliated structures, and because this combination is not observed in the other pteriomophians it has been argued to justify the ordinal level of Limida [19]. The inner aragonite shell layer of all pteriomorphians is composed of cross-lamellar crystals, with the exception of Ostreida and some Mytilida, which display nacreous tablets (Pinnoidea and Pterioidea) or in the case of Ostreoidea, a complete loss of aragonite [58,59]. In addition, the clade comprising Limida and Pectinida is the only one that combines an outer foliated calcitic structure with an inner crossed-lamellar aragonitic layer, which makes it a synapomorphy for this clade. Altogether, the distribution of this character is concordant with the classification recovered by our analyses. Interestingly, both mussels and oysters have compound latero-frontal cilia in their gills, unlike the rest of pteriomorphians [60].

### (c) Revised higher-level classification

Our results, derived from complex analyses of large datasets, and evaluating possible pitfalls affecting phylogenetic reconstruction (matrix occupancy, heterogeneity, evolutionary rates, evolutionary models) consistently recover a well-supported phylogeny of Pteriomorphia, with the only exception of the most complete (but smallest) *Matrix 3*. After exploring this potential phylogenetic inconsistency, we conclude that this result is not necessarily owing to the small matrix size, but that it may be related to the specific genes of this partition, which cause considerable conflict (electronic supplementary material, figure SA).

**Figure 4.**
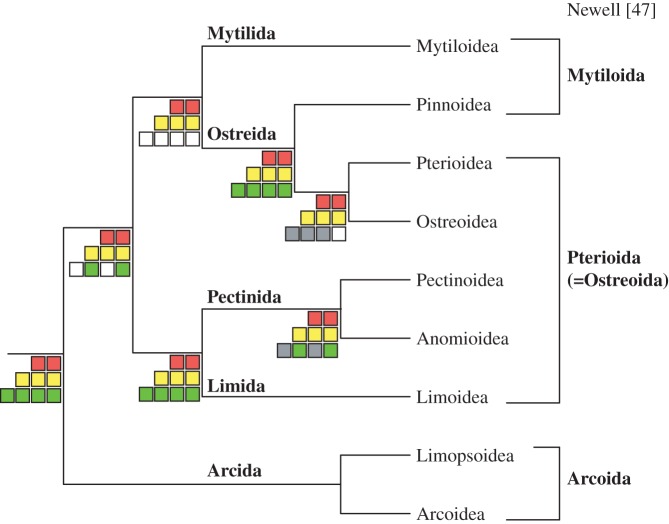
Summary tree representing relationships among the superfamilies and orders of Pteriomorphia. Checked boards are coded as in figure 2 and represent nodal support provided by *Matrices 1–3*. Newell's [47] classification is given for comparison. (Online version in colour.)

To conclude, we recognize five orders as shown in the figure 4: Mytilida (including superfamily Mytiloidea), Ostreida (including superfamilies Pinnoidea, Pterioidea, and Ostreoidea), Pectinida (including superfamilies Pectinoidea and Anomioidea), Limida (including superfamily Limoidea) and Arcida (including superfamilies Limopsoidea and Arcoidea).

## Supplementary Material

Table_S1

## Supplementary Material

Supplementary material_S2

## Supplementary Material

Figure A

## Supplementary Material

Figure B

## Data accessibility

All reads generated for this study are deposited online in the NCBI-SRA repository and all transcriptome accession numbers and museum ID are listed in the electronic supplementary material, table S1. Detailed information about the sampled specimens can be found on the MCZ online collections database: http://mczbase.mcz.harvard.edu. All in-house scripts used to construct the raw data matrices, and the raw orthogroup data matrices, are available online at https://dataverse.harvard.edu/dataverse/Pteriomorphia_phylogenomics.
