# Supplementary material for: Cementing mussels to oysters in the pteriomorphian tree: a phylogenomic approach
Source: Proc Biol Sci. 2016 Jun 29;283(1833):20160857. doi: 10.1098/rspb.2016.0857 (PMC4936043; doi:10.1098/rspb.2016.0857)
Supplement: Table_S1 [file rspb20160857supp1.pdf]

Table S1. Species sequenced and analyzed in this study, including new and publicly available data.

Order / Superfamily / Family	Species	Locality	Source	SRA #	Voucher #	Filtered Raw Reads	Contigs	N50	Filtered Peptides
**ARCIDA**									
Arcoidea									
Arcidae	*Arca noae*	Girona, Spain	González et al. 2015	SRX687762	BivAToL 116.1a	40,199,838	116,424	471	10,773
	Anadara trapezia	Queensland, Australia	Prentis & Pavasovic, 2014	SRX323049		26,285,362	91,651	631	26,912
Limopsoidea									
Philobryidae	*Neocardia* sp.	Port Elizabeth, South Africa	González et al. 2015	SRX701839	MCZ 378927	6,970,286	69,366	388	8,998
**LIMIDA**									
Limoidea									
Limidae	*Ctenoides scaber*	Bocas del Toro, Panama	*De novo*	SRR3350461	MCZ 381297	37,439,562	270,039	961	19,656
**MYTILIDA**									
Mytiloidea									
Mytilidae	*Mytilus edulis*	Massachusetts, USA	González et al. 2015	SRX687765	MCZ 381397	20,427,253	80,287	642	23,815
	Mytilus galloprovincialis	Europe	Romiguier et al. 2014	SRX565230		19,300,997	88,972	833	29,370
	Mytilus califonianus	USA	Romiguier et al. 2014	SRX565220		21,481,067	73,805	960	22,823
	Mytilus trossulus	USA	Romiguier et al. 2014	SRX565232		27,460,967	89,885	911	27,001
	Bathymodiolus azoricus	Mid Atlantic Ridge	Bettencourt et al. 2010 [2]	SRX027580		520,463	23,920	639	7,011
	Perna viridis	Hong Kong	Leung et al. 2014	SRX643380		54,132,632	47,376	1,068	15,968
**OSTREIDA**									
Ostreoidea									
Ostreidae	*Crassostrea gigas*	China	Zhang et al. 2012	Ensembl GCA_000297895.1		-	-	-	27,108
	Crassostrea virginica	China	Zhang et al. 2014	SRX118365		50,264,018	70,929	1,101	25,873
	Crassostrea hongkongensis	China	NCBI, no detailed source	SRX365659		56,540,706	78,401	920	25,177

		Crassostrea angulata		NCBI no detailed source	SRX481254		89,043	31,991	643	12,985
		Crassostrea corteziensis	Northern Mexico	NCBI no detailed source	SRX641340		2,722,278	31,129	778	11,988
		Ostrea edulis	Europe	Romiguier et al. 2014 [1]	SRX565252		21,481,067	33,186	544	6,830
		Ostrea chilensis	United Kingdom	Romiguier et al. 2014 [1]	SRX565248		3,100,945	12,390	447	3,091
		Ostrea stentina	Portugal	Romiguier et al. 2014 [1]	SRX565261		2,631,539	7,663	461	2,240
		Ostrea lurida	Washington State, USA	Timmins -Schiffman et al. 2012	SRX175407		381,100,850	7,775	495	2,072
		Saccostrea glomerata	Australia	Hook et al. 2014 [2]	SRX616573		1,100,070	26,671	756	14,201
		Saccostrea palmula	Baja California, Mexico	De novo	SRR3350462	MCZ 382150	23,847,470	22,989	373	2,002
Pinnoidea										
	Pinnidae	*Pinna saccata*	Philippines	*De novo*	SRR3350465	MCZ 381101	30,676,502	79,373	636	7,128
		Pinna atropurpurea	Philippines	*De novo*	SRR3350466	MCZ 381085	29,418,526	176,767	675	12,427
		Atrina vexillum	Moorea, French Polynesia	*De novo*	SRR3350467	MCZ 381003	42,168,232	265,939	630	30,483
		Atrina rigida	Florida, USA	González et al. 2015	SRX687763	BivAToL 14.1a	20,116,658	85,272	927	10,740
Pterioidea										
	Malleidae	*Malleus candeanus*	Bocas del Toro, Panama	*De novo*	SRR3350468	MCZ 384789	21,150,064	153,058	502	18,972
	Isognomonidae	*Isognomon alatus*	Bocas del Toro, Panama	*De novo*	SRR3350469	MCZ 381295	35,923,634	217,968	639	22,192
	Pteriidae	*Pinctada fucata*	Japan	Takeuchi et al. 2012 [1]	DRX001100		-	-	-	42,042
		Pteria colymbus	Bocas del Toro, Panama	*De novo*	SRR3350471	MCZ 381293	45,148,270	284,122	713	21,213
		Pinctada maxima	Australia	NCBI, no detailed source [2]	SRX219206		1,087,842	44,373	487	7,536
		Pinctada margaritifera	Moorea, French Polynesia	Teaniniuraitemoana et al. 2014	SRX381801		30,350,280	76,279	1,632	20,810
		Pinctada martensii	China	Zhao et al. 2012	SRX268302		13,333,138	67,631	583	23,008
**PECTINIDA**										
Anomioidea										
	Anomiidae	*Pododesmus rudis*	Bocas del Toro, Panama	*De novo*	SRR3350472	MCZ 381301	33,286,988	279,425	635	13,501
	Plicatulidae	*Plicatula plicata*	Hong Kong	*De novo*	SRR3350473	BivAToL 432.2	36,081,178	166,595	561	20,438
	Dimyidae	*Dimya lima*	Philippines	*De novo*	SRR3350463	BivAToL 177.3b	30,493,704	108,065	479	15,669
Pectinoidea										
	Pectinidae	*Placopecten magellanicus*	Massachusetts, USA	González et al. 2015	SRX687766	BivAToL 360.1a	13,721,030	30,877	913	9,736
		Azumapecten farreri		NCBI, no detailed source	SRX218546		33,879,908	80,707	514	22,969

	Mizuhopecten yessoensis	China	Ding et al. 2015	SRX483317		32,883,594	70,917	981	17,396
	Argopecten irradians	China	NCBI, no detailed source [1]	SRX470082		28,895,848	20,022	494	4,689
	Pecten maximus	Brest, France	Artigaud et al. 2014	SRX497464		28,596,492	46,866	513	5,586
	Spondylus americanus	Bocas del Toro, Panama	*De novo*	SRR3350464	MCZ 381303	42,760,882	111,336	581	6,712
**OUTGROUPS**									
**PROTOBRANCHIA**									
Solemyoidea									
Solemyidae	*Solemya velum*	Massachusetts, USA	Smith et al. 2011 [1]	SRX091478		22,761,869	98,156	2,646	16,007
Nuculoidea									
Nuculidae	*Ennucula tenuis*	Greenland	Smith et al. 2011 [1]	SRX091980		28,450,918	172,251	1,289	22,697
Nuculoidea									
Yoldiidae	*Yoldia limatula*	Massachusetts, USA	González et al. 2015	SRX687760	BivAToL 19.1a	26,199,671	15,867	521	3,671
**PALAEOHETERODONTA**									
Trigonoidea									
Trigoniidae	*Neotrigonia margaritacea*	South Australia, Australia	González et al. 2015	SRX687770	MCZ 379092	22,048,954	162,657	549	14,988
Unioniidae	*Lampsilis cardium*	Illinois, USA	González et al. 2015	SRX687767	BivAToL 421.5a	14,819,846	108,039	589	10,192
**HETERODONTA**									
Crassatelloidea									
Carditidae	*Cardites antiquata*	Spain	González et al. 2015	SRX687773	MCZ 379178	25,712,910	113,906	567	10,798
Lyonsiidae	*Lyonsia floridana*	Florida, USA	González et al. 2015	SRX687774	BivAToL 248.1a	20,343,583	92,076	838	18,084
Lucinoidea									
Lucinidae	*Phacoides pectinatus*	Florida, USA	González et al. 2015	SRX687782	BivAToL 278.1b	5,724,985	85,866	432	3,989

Note. Sequencing methods are indicated in the source column: [1] Illumina Genome Analyzer, [2] 454; all other used Illumina HiSeq. NCBI-SRA served as public archive, and Ensembl for the *Crassostrea gigas* genome. Numbered MCZ vouchers are at the Harvard Museum of Comparative Zoology; BivAToL vouchers are at the Field Museum of Natural History. Original author and date information for the included species can be found at www.molluscabase.org.

References for Table S1

1. González VL, Andrade SCS, Bieler R, Collins TM, Dunn CW, Mikkelsen PM, Taylor JD, Giribet G. 2015 A phylogenetic backbone for Bivalvia: an RNA-seq approach. *Proc. R. Soc. B* **282**, 20142332. (doi:10.1098/rspb.2014.2332).
2. Prentis PJ, Pavasovic A. 2014 The *Anadara trapezia* transcriptome: A resource for molluscan physiological genomics. *Mar. Genomics* **18**, 113-115.
3. Romiguier J, Gayral P, Ballenghien M, Bernard A, Cahais V, Chenuil A, Chiari Y, Dernat R, Duret L, Faivre N. 2014 Comparative population genomics in animals uncovers the determinants of genetic diversity. *Nature* **515**, 261-263.
4. Bettencourt R, Pinheiro M, Egas C, Gomes P, Afonso M, Shank T, Santos RS. 2010 High-throughput sequencing and analysis of the gill tissue transcriptome from the deep-sea hydrothermal vent mussel *Bathymodiolus azoricus*. *BMC Genomics* **11**, 559.
5. Leung PT, Ip JC, Mak SS, Qiu JW, Lam PK, Wong CK, Chan LL, Leung KM. 2014 De novo transcriptome analysis of *Perna viridis* highlights tissue-specific patterns for environmental studies. *BMC Genomics* **15**, 804.
6. Zhang G, Fang X, Guo X, Li L, Luo R, Xu F, Yang P, Zhang L, Wang X, Qi H. 2012 The oyster genome reveals stress adaptation and complexity of shell formation. *Nature* **490**, 49-54.
7. Timmins ‐ Schiffman EB, Friedman CS, Metzger DC, White SJ, Roberts SB. 2013 Genomic resource development for shellfish of conservation concern. *Mol. Ecology. Resour.* **13**, 295-305.
8. Hook SE, Johnston EL, Nair S, Roach AC, Moncuquet P, Twine NA, Raftos DA. 2014 Next generation sequence analysis of the transcriptome of Sydney rock oysters (*Saccostrea glomerata*) exposed to a range of environmental stressors. *Mar. Genomics* **18**, 109-111.
9. Takeuchi T, Kawashima T, Koyanagi R, Gyoja F, Tanaka M, Ikuta T, Shoguchi E, Fujiwara M, Shinzato C, Hisata K. 2012 Draft genome of the pearl oyster *Pinctada fucata*: a platform for understanding bivalve biology. *DNA Res.*, dss005.

10. Teaniniuraitemoana V, Huvet A, Levy P, Klopp C, Lhuillier E, Gaertner-Mazouni N, Gueguen Y, Le Moullac G. 2014 Gonad transcriptome analysis of pearl oyster *Pinctada margaritifera*: identification of potential sex differentiation and sex determining genes. *BMC Genomics* **15**, 491.

11. Zhao X, Wang Q, Jiao Y, Huang R, Deng Y, Wang H, Du X. 2012 Identification of genes potentially related to biomineralization and immunity by transcriptome analysis of pearl sac in pearl oyster *Pinctada martensii*. *Mar. Biotech.* **14**, 730-739.

12. Ding J, Zhao L, Chang Y, Zhao W, Du Z, Hao Z. 2015 Transcriptome Sequencing and Characterization of Japanese Scallop *Patinopecten yessoensis* from Different Shell Color Lines. *PloS One* **10**.

13. Artigaud S, Thorne MA, Richard J, Lavaud R, Jean F, Flye-Sainte-Marie J, Peck LS, Pichereau V, Clark MS. 2014 Deep sequencing of the mantle transcriptome of the great scallop Pecten maximus. *Mar. Genomics* **15**, 3-4.

14. Smith S, Wilson NG, Goetz F, Feehery C, Andrade SCS, Rouse GW, Giribet G, Dunn CW. 2011 Resolving the evolutionary relationships of molluscs with phylogenomic tools. *Nature* **480**, 364-367. (doi:10.1038/nature10526).
