# Supplementary material for: Cementing mussels to oysters in the pteriomorphian tree: a phylogenomic approach
Source: Proc Biol Sci. 2016 Jun 29;283(1833):20160857. doi: 10.1098/rspb.2016.0857 (PMC4936043; doi:10.1098/rspb.2016.0857)
Supplement: Supplementary material_S2 [file rspb20160857supp2.pdf]

Supplementary material S2

Detailed materials and methods

(a) cDNA library construction and next-generation sequencing

All cDNA libraries were constructed using the PrepX mRNA kit for Apollo 324 (Wafergen). Each library was barcoded with a TruSeq adaptor to allow multiplexed sequencing runs, with up to 6 specimens per lane. Each library concentration was measured by a real time qPCR run on a MX3000P qPCR system (Agilent Technologies) using the Kapa Library quantification kit for NGS (Kapa Biosystems); quality and size selection were assessed with an HS DNA assay in an Agilent 2100 Bioanalyzer (Agilent Technologies). Good quality libraries were then run on the Illumina HiSeq 2500 platform with paired-end reads of 150 bp at the FAS Center for Systems Biology at Harvard University.

(b) Transcriptome assembly

Demultiplexed Illumina HiSeq 2500 sequencing results were retrieved in FASTQ format from the sequencing facility (Bauer Core - Harvard University) via FTP and in SRA format from GenBank. Each sample, except for the genome of *Pinctada fucata*, was prepared as followed: Trimgalore version 0.3.3 [1], a tool incorporating both CutAdapt and FastQC, was used to quality filter the data and trim adapters. All reads with an average quality score lower than 30 based on a Phred scale, and shorter than 25 bp, were discarded. Ribosomal RNA (rRNA) was filtered out using Bowtie 2.0.0 [2] by building a bowtie index using all known mollusc rRNA sequences downloaded from GenBank. All reads that did not align with the rRNA index were stored in FASTA format as single files.

De novo assemblies were conducted for each sample with Trinity r2014-04-13 [3, 4] using paired read files and default parameters except for --path_reinforcement_distance 50, which seems to produce slightly better assemblies, with higher N50 values and longer contigs. Reduction of redundant reads was done in each transcriptome and genome with CD-HIT version 4.6 [5]using a threshold of 98% global similarity. Reduced assemblies were then processed in TransDecoder [3] to identify candidate ORFs within the transcripts. Predicted peptides were filtered for isoforms by selecting only one peptide per putative

unigene with a custom Python script, thus removing the variation in the coding regions of Trinity assemblies due to alternative splicing, closely related paralogs, and allelic diversity. Filtered peptide sequences with all final candidate ORFs were retained as multifasta files. Asterisks, indicating stop codons, as well as blank lines between individual contigs, were deleted from each file.

(c) Orthology assignment and matrix construction

Orthology assignment for the data set assemblies was performed using stand-alone OMA v0.99z.2 [6, 7]. The parameters.drw file specified retained all default settings with the exception of "MaxTimePerLevel," which was set at 3600. The all-by-all local alignment process was parallelized across 128 CPUs once all the input pre-processing steps were achieved on a single core (to avoid risk of collision).

The orthogroup selection based on minimum taxon occupancy was executed using a custom Python script. Selected orthogroups for each matrix were aligned individually using MUSCLE version 3.6 [8]. Divergently aligned positions were culled by a probabilistic character masking approach with ZORRO [9], using default parameters and FastTree 2.1.4 [10] to construct guide trees. In all of the alignments, positions that were assigned a confidence score below the threshold of 5 by ZORRO were discarded, using a custom Python script. Trimmed orthogroups were then concatenated using Phyutility 2.6 [11].

In order to assess the effects of rate of molecular evolution and heterotachy on tree topology ten additional matrices were constructed by selecting subsets of *Matrix 2* based on evolutionary rate, for which percent pairwise identity was employed as a proxy. Accumulated conservation values were generated for each locus using Trimal 1.2b (-sct flag). Values were then normalized to account for length. Loci were sorted; the first being the slowest evolving genes (most conserved) and the last being the fastest evolving genes (least conserved). Matrices were produced as follows: 1) *Matrices A-D* were constructed with incremental addition of loci to produce four matrices with 25 (*Matrix A*), 50 (*Matrix B*), 100 (*Matrix C*), 200 (*Matrix D*), and 277 loci (same as *Matrix 2*) total. This strategy has been used in previous phylogenomic analyses [12-14], but masks the possible contribution of the fastest evolving genes, as the slowest ones are always used. In order to investigate the contribution of the different blocks of evolutionary rates in absence of the other genes, we

designed another strategy: *Matrices E-J* were constructed by parsing 50 loci matrices and one 27 loci matrix, with no addition (*see* figure 1). The percent pairwise identity was calculated for each of the 277 orthogroup alignments subsequent to treatment with GBlocks v. 0.91b [15]. This ensured that regions with large indels did not affect the calculation of percent pairwise identity.

The package BaCoCa v.1.1r was used to estimate relative composition frequency variability (RCFV) in *Matrix 2*. RCFV is a measure of the absolute deviation from the mean for each amino acid and for each taxon summed up over all amino acids and all taxa [16]. The higher the RCFV value, the higher the degree of compositional heterogeneity present in that partition. RCFV values were plotted in a heatmap using the R package gplots.

(d) Phylogenetic analyses

Matrix 1 was analyzed with ExaML v. 3.0 [17] with the per-site rate category (PSR) model. Bootstrap resampling was conducted for 100 independent replicates using RAxML v. 8.2 [18], specifying a model of protein evolution with corrections for a discrete gamma distribution using the WAG model, and were thereafter mapped onto the optimal tree from the independent ExaML searches.

The initial three matrices (*Matrices 1*, *2* and *3*) were analyzed using Bayesian inference with ExaBayes version 1.21 with openmpi version 1.64. ExaBayes uses a sampling approach similar to the one implemented in MrBayes [19], but it is better adapted for large data sets by its ability to parallelize each independent run, each chain, and the data (i.e., unique site patterns of the alignment). ExaBayes implements a Markov chain Monte Carlo (MCMC) sampling approach. We used the amino acid model prior (aaPR), a discrete model prior, which mixes a combination of 18 models of evolution. Four independent Markov chain Monte Carlo chains (MCMC) were run for 1,000,000 generations, sampling every 500th generation. The first 2,000 trees (25%) were discarded as burn-in for each MCMC run prior to convergence (i.e., when maximum discrepancies across chains < 0.1). Convergence of parameters between runs was estimated using the command *postProcParam* available in exabayes software. Most parameters showed convergence except for the analysis conducted on *Matrix 1*. Trees from all runs were combined after burn-in to obtain the consensus

majority rule tree topology and node marginal probability (i.e. posterior credibility values of node or confidence support) with the command *consense* implemented in ExaBayes.

To test for putative gene incongruence, we inferred individual gene trees for each orthogroup included in each of the three initial matrices (*Matrices 1*, *2* and *3*) using RAxML 7.7.5 [20]. PROTGAMMALG4X was selected as the best model of aa substitution. All individual best-scoring trees were concatenated for each matrix and fed into SuperQ v1.1 [21] in order to visualize inter-gene conflicts. SuperQ decomposes all gene trees into quartets to infer a super-network where edge lengths are assigned based on quartet frequencies; it was run using the 'balanced' edge-weight optimization function with no filter. The resulting super-networks were visualized with SplitsTree v.4.13.1 [22].

References for Supplementary Material S2

1. Wu ZP, Wang X, Zhang XG. 2011 Using non-uniform read distribution models to improve isoform expression inference in RNA-Seq. *Bioinformatics* **27**, 502-508. (doi:10.1093/Bioinformatics/Btq696).
2. Langmead B, Trapnell C, Pop M, Salzberg SL. 2009 Ultrafast and memory-efficient alignment of short DNA sequences to the human genome. *Genome Biol.* **10**, R25. (doi:10.1186/gb-2009-10-3-r25).
3. Haas BJ, Papanicolaou A, Yassour M, Grabherr M, Blood PD, Bowden J, Couger MB, Eccles D, Li B, Lieber M, et al. 2013 *De novo* transcript sequence reconstruction from RNA-seq using the Trinity platform for reference generation and analysis. *Nat. Protocols* **8**, 1494-1512. (doi:10.1038/nprot.2013.084).
4. Grabherr MG, Haas BJ, Yassour M, Levin JZ, Thompson DA, Amit I, Adiconis X, Fan L, Raychowdhury R, Zeng QD, et al. 2011 Full-length transcriptome assembly from RNA-Seq data without a reference genome. *Nat. Biotechnol* **29**, 644-652. (doi:10.1038/Nbt.1883).

5. Fu LM, Niu BF, Zhu ZW, Wu ST, Li WZ. 2012 CD-HIT: accelerated for clustering the next-generation sequencing data. *Bioinformatics* **28**, 3150-3152. (doi:10.1093/Bioinformatics/Bts565).
6. Altenhoff AM, Gil M, Gonnet GH, Dessimoz C. 2013 Inferring hierarchical orthologous groups from orthologous gene pairs. *PLoS ONE* **8**, e53786. (doi:10.1371/journal.pone.0053786).
7. Altenhoff AM, Schneider A, Gonnet GH, Dessimoz C. 2011 OMA 2011: orthology inference among 1000 complete genomes. *Nucleic Acids Res.* **39**, D289-D294. (doi:10.1093/Nar/Gkq1238).
8. Edgar RC. 2004 MUSCLE: multiple sequence alignment with high accuracy and high throughput. *Nucleic Acids Res.* **32**, 1792-1797.
9. Wu M, Chatterji S, Eisen JA. 2012 Accounting for alignment uncertainty in phylogenomics. *PLoS ONE* **7**, e30288. (doi:10.1371/journal.pone.0030288).
10. Price MN, Dehal PS, Arkin AP. 2010 FastTree 2-approximately maximum-likelihood trees for large alignments. *PLoS ONE* **5**, e9490. (doi:10.1371/journal.pone.0009490).
11. Smith SA, Dunn CW. 2008 Phyutility: a phyloinformatics tool for trees, alignments and molecular data. *Bioinformatics* **24**, 715-716. (doi:10.1093/bioinformatics/btm619).
12. Fernández R, Hormiga G, Giribet G. 2014 Phylogenomic analysis of spiders reveals nonmonophyly of orb weavers. *Curr. Biol. : CB* **24**, 1772-1777. (doi:10.1016/j.cub.2014.06.035).
13. Sharma PP, Kaluziak S, Pérez-Porro AR, González VL, Hormiga G, Wheeler WC, Giribet G. 2014 Phylogenomic interrogation of Arachnida reveals systemic conflicts in phylogenetic signal. *Mol. Biol. Evol.* **31**, 2963-2984. (doi:10.1093/molbev/msu235).
14. Andrade SCS, Novo M, Kawauchi GY, Worsaae K, Pleijel F, Giribet G, Rouse GW. 2015 Articulating “archiannelids”: Phylogenomics and annelid relationships, with emphasis on meiofaunal taxa. *Mol. Biol. Evol.* **32**, 2860-2875. (doi:10.1093/molbev/msv157).
15. Castresana J. 2000 Selection of conserved blocks from multiple alignments for their use in phylogenetic analysis. *Mol. Biol. Evol.* **17**, 540-552.

16. Kück P, Struck TH. 2014 BaCoCa–A heuristic software tool for the parallel assessment of sequence biases in hundreds of gene and taxon partitions. *Mol. Phylogenet. Evol.* **70**, 94-98.
17. Kozlov AM, Aberer AJ, Stamatakis A. 2015 ExaML version 3: a tool for phylogenomic analyses on supercomputers. *Bioinformatics* **31**, 2577-2579. (doi:10.1093/bioinformatics/btv184).
18. Stamatakis A. 2014 RAxML version 8: A tool for phylogenetic analysis and post-analysis of large phylogenies. *Bioinformatics* **30**, 1312-1313. (doi:10.1093/bioinformatics/btu033).
19. Ronquist F, Huelsenbeck JP. 2003 MrBayes 3: Bayesian phylogenetic inference under mixed models. *Bioinformatics* **19**, 1572-1574.
20. Berger SA, Krompass D, Stamatakis A. 2011 Performance, accuracy, and Web server for evolutionary placement of short sequence reads under maximum likelihood. *Syst. Biol.* **60**, 291-302. (doi:10.1093/sysbio/syr010).
21. Grünewald S, Spillner A, Bastkowski S, Bogershausen A, Moulton V. 2013 SuperQ: computing supernetworks from quartets. *IEEE/ACM Trans. Comput. Biol. Bioinform.* **10**, 151-160. (doi:10.1109/TCBB.2013.8).
22. Huson DH, Bryant D. 2006 Application of phylogenetic networks in evolutionary studies. *Mol. Biol. Evol.* **23**, 254-267. (doi:10.1093/molbev/msj030).
