# Supplementary figures and images for: Cementing mussels to oysters in the pteriomorphian tree: a phylogenomic approach
Source: Proc Biol Sci. 2016 Jun 29;283(1833):20160857. doi: 10.1098/rspb.2016.0857 (PMC4936043; doi:10.1098/rspb.2016.0857)

*Matrix 1*

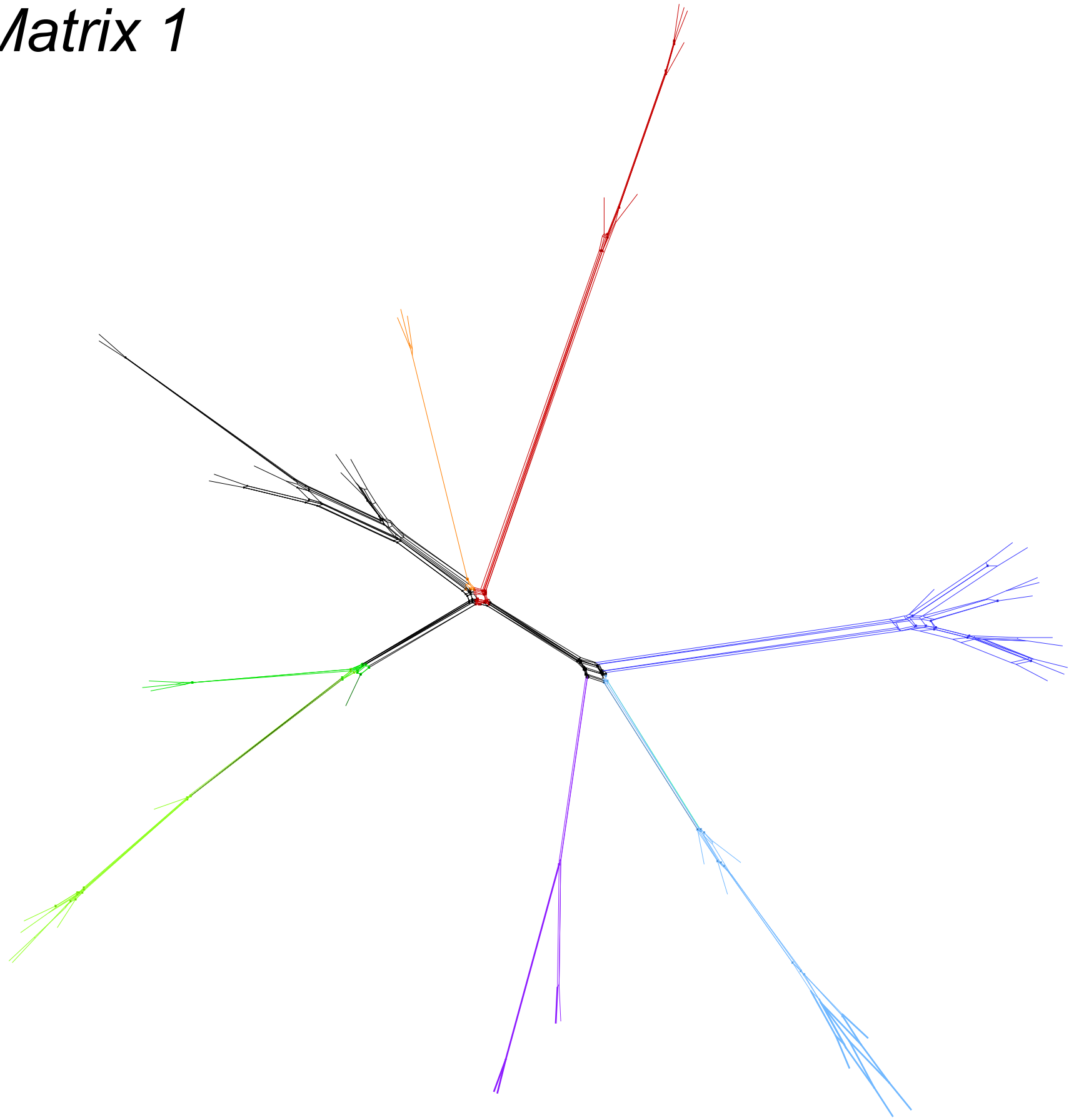

*Matrix 3*

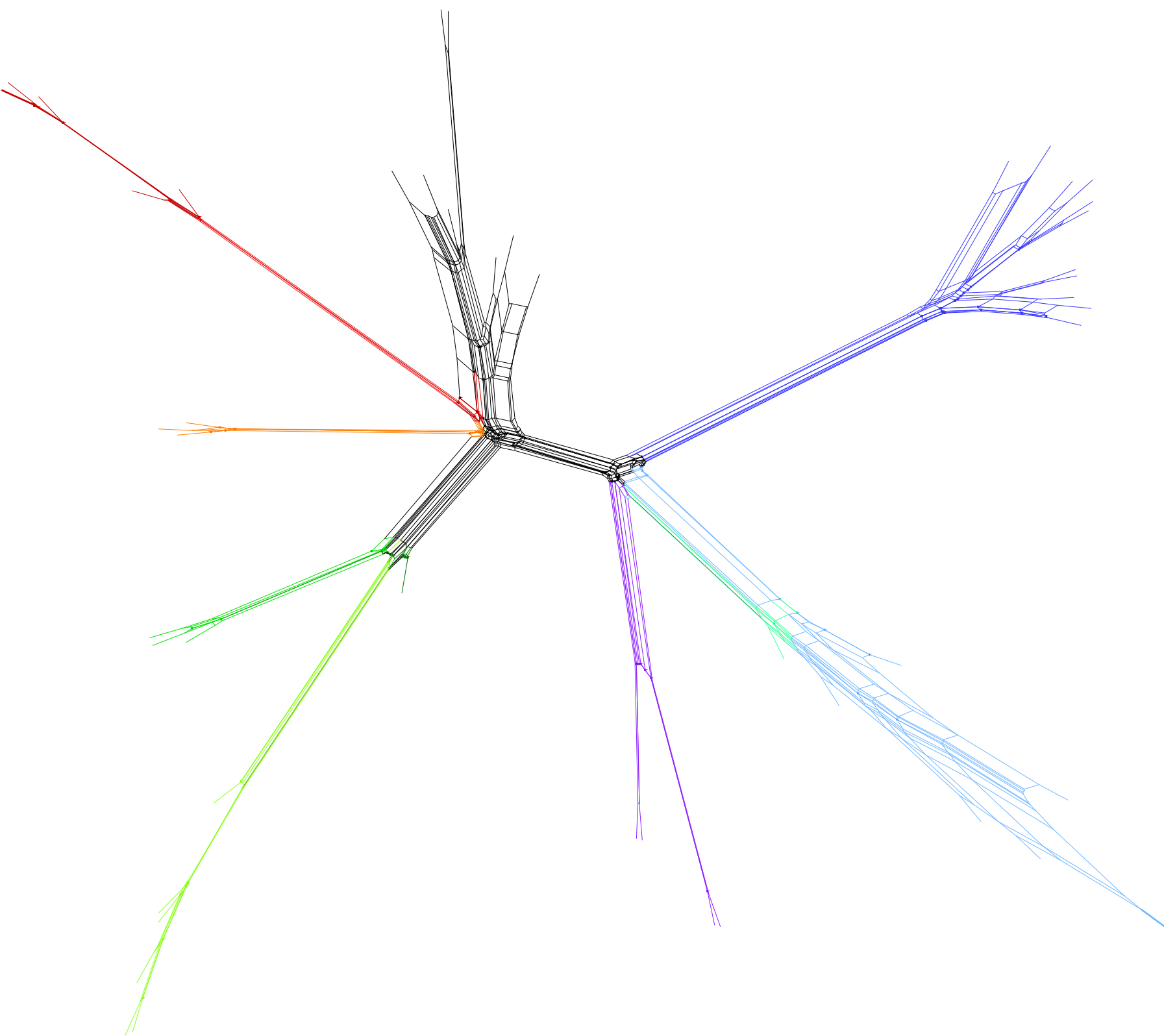

Supplement: Figure A [file rspb20160857supp3.pdf]
