# Supplementary material for: Cementing mussels to oysters in the pteriomorphian tree: a phylogenomic approach
Source: Proc Biol Sci. 2016 Jun 29;283(1833):20160857. doi: 10.1098/rspb.2016.0857 (PMC4936043; doi:10.1098/rspb.2016.0857)
Supplement: Figure B [file rspb20160857supp4.pdf]

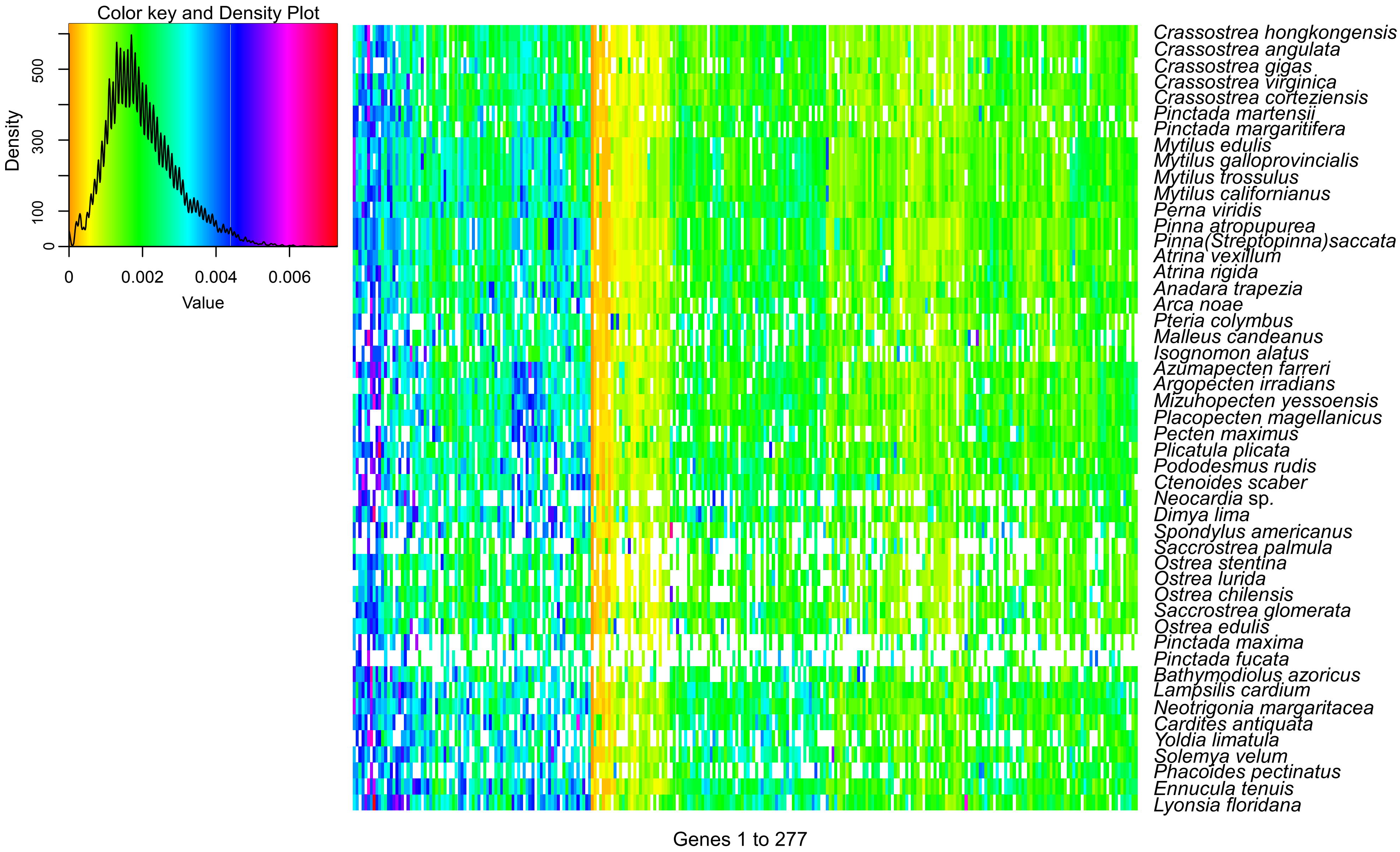
Color key and Density Plot
Density
0
100
300
500
0
0.002
0.004
0.006
Value
Crassostrea hongkongensis
Crassostrea angulata
Crassostrea gigas
Crassostrea virginica
Crassostrea corteziensis
Pinctada martensii
Pinctada margaritifera
Mytilus edulis
Mytilus galloprovincialis
Mytilus trossulus
Mytilus californianus
Perna viridis
Pinna atropupurea
Pinna(Streptopinna)saccata
Atrina vexillum
Atrina rigida
Anadara trapezia
Arca noae
Pteria colymbus
Malleus candeanus
Isognomon alatus
Azumapecten farreri
Argopecten irradians
Mizuhopecten yessoensis
Placopecten magellanicus
Pecten maximus
Plicatula plicata
Pododesmus rudis
Ctenoides scaber
Neocardia sp.
Dimya lima
Spondylus americanus
Saccrostrea palmula
Ostrea stentina
Ostrea lurida
Ostrea chilensis
Saccrostrea glomerata
Ostrea edulis
Pinctada maxima
Pinctada fucata
Bathymodiolus azoricus
Lampsilis cardium
Neotrigonia margaritacea
Cardites antiquata
Yoldia limatula
Solemya velum
Phacoides pectinatus
Ennucula tenuis
Lyonsia floridana
Genes 1 to 277
